# A comparison of Indigenous and non-Indigenous survivors of sexual assault and their receipt of and satisfaction with specialized health care services

**DOI:** 10.1371/journal.pone.0188253

**Published:** 2017-11-16

**Authors:** Janice Du Mont, Daisy Kosa, Sheila Macdonald, Anita Benoit, Tonia Forte

**Affiliations:** 1 Women’s College Research Institute, Women’s College Hospital, Toronto, Ontario, Canada; 2 Dalla Lana School of Public Health, University of Toronto, Toronto, Ontario, Canada; 3 Ontario Network of Sexual Assault/Domestic Violence Treatments Centres, Toronto, Ontario, Canada; 4 Waakebiness-Bryce Institute for Indigenous Health, University of Toronto, Toronto, Ontario, Canada; Royal Children's Hospital, AUSTRALIA

## Abstract

In Canada, Indigenous women are more likely than non-Indigenous women to be survivors of sexual assault and experience sexual assaults that are more serious in terms of physical injury and other health-related consequences. Despite their related needs for care and support, there is a paucity of research to date that has examined their uptake of specialized acute health services post sexual assault. To address this gap, we explored the presentation, sociodemographic, assailant, assault, and service use characteristics of Indigenous women, as compared to non-Indigenous adult and adolescent women aged 12 and older presenting to 30 of 35 hospital-based sexual assault treatment centres in Ontario from 2009 to 2011, using bivariate analyses. Of the 948 women in our sample, 116 (12%) identified as being Indigenous. Indigenous survivors differed significantly from non-Indigenous survivors on many presentation, sociodemographic, and assault characteristics. For example, they were more likely to present to a hospital within 24 hours of being assaulted and a treatment centre serving a primarily rural population. They tended to be younger, were more likely to be living in an institutional setting, report community or group affiliations and government or community services as sources of social support, and be assaulted by a parent, guardian, or other relative. In terms of receipt of services, they were more likely to have undergone safety planning and to be referred to child protection or community agencies. They reported high levels of satisfaction with the services received, however, were less likely than non-Indigenous survivors to rate the overall care provided as excellent or good. On the whole, the results of our study suggest that Indigenous women value acute hospital-based sexual assault services. However, they experience sexual assaults in contexts different from non-Indigenous survivors. It is important for health care providers to be attuned to this so that they can appropriately respond to their unique needs.

## Introduction

In Canada, there is a long history of the marginalization and oppression of Indigenous people, including the expropriation of Indigenous lands, relocation of Indigenous communities to reservations and Indigenous children to residential schools and to non-Indigenous families, and prohibition against Indigenous religious and spiritual practices [1, 2]. Some researchers theorize that this traumatic history has contributed to the magnitude and severity of the current problem of violence against Indigenous women in Canada [3]. Indigenous women in Canada are disproportionately affected by violence being 2.6 times more likely than non-Indigenous women to report being a victim of a violent crime [4]. Indigenous women in Canada are also more likely than non-Indigenous women to experience one of the most pernicious form of violence against women, sexual assault, at a rate more than three times higher than non-Indigenous women (115 incidents per 1,000 vs. 35 per 1,000) [5].

Some previous research has shown that Indigenous women experience severer sexual assaults than non-Indigenous women. Analyses of data from 1992–2005 of the National Crime Victimization Survey, a nationally representative survey which monitors rape and sexual assault on an annual basis in the United States, found that sexually assaulted American Indian and Alaskan Native women were more likely to be hit (91%), injured (20%), and to face an armed offender (25%) than African American (78%, 16%, 9%, respectively) and White (71%, 16%, 9%, respectively) women [6]. Further analyses of this survey revealed that among women who sustained injuries from an assault, American Indian and Alaska Native women were more likely to require medical care for their injuries as a result of being attacked (47%) as compared to African American (35%) and White women (33%) [6]. These findings are consistent with analyses of Statistics Canada data that have shown that Indigenous survivors are more likely than non-indigenous survivors of intimate partner abuse to experience the most serious forms violence, including having been sexually assaulted, strangled, beaten, and threatened with a weapon, and to be injured as a result [4].

A qualitative study by Dylan, Regehr, and Allagia found that Indigenous sexual assault survivors’ interactions with health and social service providers were supportive and, as a result, helped facilitate their healing [7]. However, victimized Indigenous women often face unique or enhanced barriers in accessing health services related to language, health literacy, values, and culture; fear of experiencing racism, victim blaming, prejudice or other unfair treatment, and loss of children to authorities; and geographic isolation [8–12]. Even when they are able to and do access services, providers must be cognizant that Indigenous women experience sexual assault and other forms of abuse disproportionately or differently due to social and historical factors that may impact the ways in which services should be offered and delivered. Such factors include racism, cultural devaluation, social and economic exclusion, and colonization [13–15].

Amnesty International (2007) has recommended that health and other services work collaboratively to streamline and optimize care in a manner sensitive to the needs of Indigenous survivors, while also addressing the barriers to service use this population commonly faces [16]. This non-governmental organization specifically has called for the further implementation of hospital-based violence treatment programs, led by specially trained health care providers such as sexual assault nurse examiners (SANEs), to better serve sexually assaulted Indigenous women as, such “[h]ealth service providers have a key role to play both in providing survivors with any medical attention they may need and in documenting sexual violence” ([16], see p. 9).

Hospital-based violence treatment programs are designed to address the acute needs of survivors of sexual assault by offering emergency medical care, including treatment for any injuries sustained, treatment for sexually transmitted infections, and provision of emergency contraception. In addition, forensic evidence is collected including the documentation of injuries and collection of biological samples for toxicology testing and swabs for testing for presence of DNA, using a sexual assault or rape evidence collection kits. These treatment programs are also aimed at minimizing the consequences of abuse and likelihood of re-victimization through the offering of risk assessment, safety planning, and follow-up/referral services [17, 18].

Some studies have been conducted examining the uptake and satisfaction with SANE-led violence treatment services among all clients [17, 19]. However, there is limited research that has focused specifically on Indigenous women, and compared their experiences to those of non-Indigenous survivors seen at such services. This is a critical gap in knowledge as women are not a monolithic group, and explorations of diverse groups within the category of “women” can reveal important differences, which may have implications for health policy and the delivery of health care [20, 21]. To address this gap, in this study, we examined data drawn from a client evaluation of services conducted at Ontario’s Sexual Assault/Domestic Violence Treatment Centres (SA/DVTCs) [19]. Although the Client Evaluation Project was not designed specifically to examine the experiences of Indigenous women and girls, the data collected provided an important opportunity to explore the presentation, sociodemographic, assailant, assault, and service use characteristics of Indigenous women, as compared to non-Indigenous survivors. The results of this study hold the potential to inform outreach initiatives and systems of health service delivery to Indigenous survivors, as well as further training for post-sexual assault service providers.

## Methods

### Ethics

We obtained research ethics board approval from Women’s College Hospital (REB: 2016-0028-E) for the current study. For the Client Evaluation Project ethics approval was attained at all 30 participating hospital-based SA/DVTCs. As part of the research ethics process, informed consent was obtained from each participant or parent/guardian.

### Data collection tools

Two data collection forms [19], designed by the research team of experienced SANEs and gender-based violence researchers, were developed based on information collected in previous sexual assault studies [22–28] and SA/DVTC standards of care [29]. These forms were piloted at two sites for several weeks and modified based on feedback. The resulting standardized client intake form and the satisfaction survey collected the following information examined in this study (see Tables for full list of variables): *presentation characteristics* such as police accompaniment; *sociodemographic characteristics* such as age, race/ethnicity, marital status, disability status; *assailant characteristics* such as relationship to survivor; and *assault characteristics* such as type of sexual assault, number of sex acts, and type of coercion. Additionally, information was examined about *receipt of services* including assessment/documentation of injuries, photodocumentation of injuries, crisis intervention/counselling, medical care and/or treatment, risk assessment, safety planning, and referral to community agencies for ongoing support. Finally, information about *satisfaction with services* was examined including timely response to needs, felt safe during visit, all questions and concerns responded to, was treated with respect, was supported and cared for, was not judged (strongly agree/agree/disagree/strongly disagree), needed care was received (yes/no), and overall rating of care (excellent/good/fair/poor).

### Procedure

In the Client Evaluation Project, data were collected prospectively between April 1, 2009 and June 30, 2011 at 30 of Ontario’s 35 participating SA/DVTCs. To ensure that the data were collected in a standardized manner across all sites, regional train-the-trainer sessions were delivered to managers of each program by one of the study principal investigators, also an experienced SANE (SM). The program managers in turn trained their own frontline nurses.

All clients who are survivors of sexual assault or domestic violence who presented within approximately 72 hours of a sexual assault were interviewed by attending SANEs and offered services as appropriate as part of the delivery of clinical care. Clients were free to accept or decline any services. The satisfaction survey, which was provided to the client or parent/guardian by the attending SANE who explained its voluntary and anonymous nature, was completed after receipt of care either in a private area on site or at home (provided with a self-addressed, stamped, envelope). Both the client intake form and satisfaction surveys were linked only with a unique study ID number for each client. The intake and satisfaction forms were sent to the central coordinating centre where the information was entered into a secure database on an ongoing basis.

### Analysis

The analyses and interpretation of the data for the current study were guided by our Indigenous research team member (AB). The analyses included adult and adolescent women 12 years of age and older who were sexually assaulted and identified their ethnicity. We compared those who identified as Indigenous (e.g., Aboriginal, First Nations, Métis) to women and girls who identified as other ethnic/racial groups. We used Pearson’s Chi-square or Fisher’s Exact tests where appropriate to examine associations between Indigenous status and presentation, sociodemographic, assailant, and assault characteristics, as well as receipt of and satisfaction with services. For satisfaction with services, the decision to examine response options as strongly agree/agree vs. disagree/strongly disagree and excellent/good versus fair/poor was made a priori to conducting the analyses for this study, and is consistent with other analyses arising from the larger Client Evaluation Project [19].

For the number and type of sex acts, survivors indicating “don’t know/remember” ranged from 37%-45% for Indigenous women and 32%-41% for non-Indigenous women and these cases were therefore included in the relevant analyses. Missing data on other individual variables examined were excluded from the analyses. If cell counts were less than 5, data were suppressed to be sensitive to concerns relating to confidentiality. Complementary cells were also suppressed where the data contained in those cells could be used to compute those with cell counts less than 5 [30]. All analyses were conducted using Stata Statistical software (StataCorp, 2001) and a *p* value < 0.05 was used to indicate statistical significance, as is appropriate given the exploratory nature of the study [31].

## Results

Nine hundred forty eight clients met study inclusion criteria—were adult and adolescent women aged 12 years or older, presented for acute sexual assault care, and identified their ethnicity. Of these, 116 (12.2%) identified as Indigenous and the remaining 832 (87.8%) as non-Indigenous. Seventy-two Indigenous survivors (62.1%) and 596 (71.6%) non-Indigenous survivors had filled in a satisfaction survey.

### Presentation characteristics

Indigenous adult and adolescent women differed significantly from non-Indigenous survivors on all presentation characteristics (Table 1). Indigenous women were more likely to be accompanied to the hospital by police (51.4% vs. 39.3%, p = 0.049). They were also more likely to present to a hospital within 24 hours of being sexually assaulted (69.6% vs. 58.8%, p = 0.028) and at a treatment centre serving a primarily rural population (53.1% vs. 17.2%, p<0.0001).

**Table 1:** Presentation characteristics of sexually assaulted adolescent and adult women who presented to Sexual Assault/Domestic Violence Treatment Centres, by Indigenous status.

Characteristics	Indigenous	Non-Indigenous	p-value
Police accompaniment	**n = 72 (%)**	**n = 596 (%)**	0.049
	37 (51.4)	232 (39.3)	
Time from assault to initial presentation at hospital	**n = 116 (%)**	**n = 832 (%)**	0.028
Less than 24 hours	78 (69.6)	462 (58.8)	
24 hours or more	34 (30.4)	324 (41.2)	
Population served primarily by centre	**n = 116 (%)**	**n = 832 (%)**	<0.0001
Rural	60 (53.1)	140 (17.2)	
Suburban/urban	53 (46.9)	673 (82.8)	

### Sociodemographic characteristics

Indigenous adult and adolescent women differed significantly from non-Indigenous survivors on several sociodemographic characteristics (Table 2). Indigenous women experiencing sexual assault were more likely to be between 12 to 18 years of age at the time of the assault (43.5% vs. 31.2%, p = 0.008). Indigenous survivors were more likely to live in an institutional setting at the time of the assault (11.2% vs. 3.4%, p<0.0001), and less likely to live with a roommate or in a dormitory (7.8% vs. 16.6%, p = 0.014). They were also more likely to report community or group affiliations (25.9% vs. 14.8%, p = 0.002) and government or community services (9.5% vs. 3.6%, p = 0.004) as sources of social support, and less likely to report roommates or friends (43.1% vs. 64.4%, p<0.0001) and school personnel (9.5% vs. 16.8%, p = 0.043) as supportive.

**Table 2:** Sociodemographic characteristics of sexually assaulted adolescent and adult women who presented to Sexual Assault/Domestic Violence Treatment Centres, by Indigenous status.

Characteristics	Indigenous n = 116 (%)	Non-Indigenous n = 832 (%)	p-value
Age (years)			0.008
12 to 18	50 (43.5)	257 (31.2)	
19 and older	65 (56.5)	567 (68.8)	
Marital status			0.50
Never married	83 (73.5)	634 (77.4)	
Separated/divorced/widowed	12 (10.6)	86 (10.5)	
Married/common law/cohabitating	18 (15.9)	99 (12.1)	
Disability			0.25
Yes	25 (21.6)	143 (17.2)	
No	91 (78.5)	689 (82.8)	
Employment status			<0.0001
Employed	17 (15.9)	339 (42.1)	
Not employed	90 (84.1)	467 (57.9)	
Student			0.82
Yes	50 (46.7)	367 (45.5)	
No	57 (53.3)	439 (54.5)	
Living situation*			
Lives alone	17 (14.7)	150 (18.0)	0.372
With family	82 (70.7)	550 (66.2)	0.335
In institutional setting**	13 (11.2)	28 (3.4)	<0.0001
Homeless/in shelter	7 (6.0)	41 (4.9)	0.61
With roommate/in dormitory	9 (7.8)	138 (16.6)	0.014
Social supports*			
None	6 (5.2)	64 (7.7)	0.33
Family	94 (81.0)	677 (81.4)	0.93
Roommate/friend	50 (43.1)	536 (64.4)	<0.0001
Community/group affiliation	30 (25.9)	123 (14.8)	0.002
School personnel	11 (9.5)	140 (16.8)	0.043
Mental health professional	6 (5.2)	46 (5.5)	0.87
Government/community services	11 (9.5)	30 (3.6)	0.004
Other (e.g., physician, nurse, police)	--	6 (0.7)	0.60
Unknown	--	7 (0.8)	0.30

*Women could indicate more than one response.

**Includes, for example, foster care, group home, long-term care.

--Data suppressed due to small cell size.

### Assailant characteristics

Indigenous adult and adolescent women differed significantly from non-Indigenous survivors on their relationship to the assailant (Table 3). In cases in which there was only one assailant, Indigenous women were more likely to have been assaulted by a parent, guardian, or other relative (14.8% vs. 3.1%), less likely by a partner or ex-partner (13.6% vs. 19.2%) and other known assailant (54.3% vs. 61.1%), and as likely by a stranger (17.3% vs. 16.6%, p <0.0001).

**Table 3:** Assailant characteristics of sexually assaulted adolescent and adult women who presented to Sexual Assault/Domestic Violence Treatment Centres, by Indigenous status.

Characteristics	Indigenous n = 116 (%)	Non-Indigenous n = 832 (%)	p-value
Number of assailants			0.939
1	84 (75.7)	586 (74.8)	
2 or more	14 (12.6)	96 (12.3)	
Don’t know/remember	13 (11.7)	101 (12.9)	
Relationship to assailant*			<0.0001
Partner/ex-partner	11 (13.6)	111 (19.2)	
Parent/guardian/other relative	12 (14.8)	18 (3.1)	
Other known assailant	44 (54.3)	354 (61.1)	
Stranger	14 (17.3)	96 (16.6)	

*Among those survivors who reported one assailant.

### Assault characteristics

Indigenous adult and adolescent women differed significantly from non-Indigenous survivors on a few assault characteristics (Table 4). Indigenous women were less likely to report any other coercion, that is, coercion not involving physical or psychological force (15.8% vs. 25.2%, p = 0.028) and, specifically, having been forced to drink or having been drugged prior to being assaulted (5.3% vs. 11.9%, p = 0.034).

**Table 4:** Assault characteristics of sexually assaulted adolescent and adult women who presented to Sexual Assault/Domestic Violence Treatment Centres, by Indigenous status.

Characteristics	Indigenous n = 116 (%)	Non-Indigenous n = 832 (%)	p-value
Weapon			0.803
Yes	--	34 (4.4)	
No	83 (76.9)	575 (73.9)	
Don’t know/remember	--*	169 (21.7)	
Types of coercion*			
*Any physical*	58 (50.8)	369 (46.3)	0.360
Restrained	36 (31.6)	258 (32.4)	0.866
Pushed	32 (28.1)	205 (25.7)	0.593
Slapped/kicked/bit/hair pulled	17 (14.9)	111 (13.9)	0.777
Beaten	--	23 (2.9)	0.587
Strangled	9 (7.9)	53 (6.7)	0.622
Stabbed	--	--	0.765
*Any psychological*	23 (20.2)	197 (24.8)	0.283
Threatened	19 (16.7)	135 (17.0)	0.933
Manipulated	9 (7.9)	84 (10.5)	0.383
*Any other coercion*	18 (15.8)	201 (25.2)	0.028
Forced to drink/drugged	6 (5.3)	95 (11.9)	0.034
Sleeping/unconscious	12 (10.5)	136 (17.1)	0.077
Physical injuries	40 (37.4)	288 (37.8)	0.927
Injury type			0.685
Genital	7 (22.6)	76 (29.8)	
Extra-genital	18 (58.1)	138 (54.1)	
Both	6 (19.4)	41 (16.1)	
Types of sex acts*			
Touching/fondling	41 (36.6)	345 (45.5)	0.196
Cunnilingus	11 (9.9)	91 (12.4)	0.742
Fellatio	15 (13.2)	148 (18.6)	0.159
*Any vaginal penetration*	60 (55.1)	491 (65.6)	0.080
Vaginal penetration: finger	22 (20.4)	223 (29.3)	0.140
Vaginal penetration: penis	55 (49.6)	448 (58.6)	0.199
Vaginal penetration: foreign object	--	21 (2.8)	0.597
*Any anal penetration*	22 (20.8)	110 (16.0)	0.179
Anal penetration: finger	9 (8.3)	41 (5.5)	0.377
Anal penetration: penis	17 (15.5)	90 (12.2)	0.340
Anal penetration: foreign object	--	11 (1.5)	0.332
Number of sex acts			0.330
1	37 (33.3)	273 (36.1)	
2 to 5	20 (18.0)	175 (23.1)	
6 or more	--	15 (2.0)	
Don’t know/remember	--*	294 (38.8)	

* Women could indicate more than one response.

--Data suppressed due to small cell size.

--*complementary cell suppression.

### Receipt of services

Indigenous adult and adolescent women differed significantly from non-Indigenous survivors in their receipt of some SA/DVTC services (Table 5). Indigenous women were more likely to receive risk assessment or safety planning overall (67.5% vs. 54.6%; p = 0.009). With respect to specific services used, Indigenous women were more likely to receive safety planning support (51.8% vs. 39.7%, p = 0.014) and be referred to a child protection agency (14.0% vs. 4.9%, p<0.0001). They were also more likely to be referred to community agencies for ongoing support following sexual assault (44.7% vs. 32.4%, p = 0.009).

**Table 5:** Receipt of services of sexually assaulted adolescent and adult women who presented to Sexual Assault/Domestic Violence Treatment Centres, by Indigenous status.

Characteristics	Indigenous n = 116 (%)	Non-Indigenous n = 832 (%)	p-value
*Any forensic evaluation*	103 (90.4)	709 (89.0)	0.655
Sexual Assault Evidence Kit completion	68 (59.7)	481 (60.4)	0.886
Assessment/documentation of injuries	96 (84.2)	669 (83.9)	0.941
Photodocumentation of injuries	22 (19.3)	138 (17.3)	0.603
Anal/rectal examination	28 (24.6)	181 (22.7)	0.660
Colposcope/medscope examination	--	19 (2.4)	0.304
Vaginal examination with speculum	47 (41.2)	396 (49.7)	0.091
*Any acute health care*	107 (93.9)	749 (94.0)	0.961
Medical care/treatment	83 (72.8)	563 (70.6)	0.634
Prophylaxis for treatment of STIs	87 (76.3)	594 (74.5)	0.681
Emergency contraception	62 (54.4)	443 (55.6)	0.810
HIV PEP counselling	74 (64.9)	515 (64.6)	0.951
Crisis counselling	73 (64.0)	506 (63.5)	0.910
*Any risk assessment or safety planning*	77 (67.5)	435 (54.6)	0.009
Risk assessment	56 (49.1)	332 (41.7)	0.132
Safety planning	59 (51.8)	316 (39.7)	0.014
Referral to child protection agency	16 (14.0)	39 (4.9)	<0.0001
*Any referral to services for ongoing support*	93 (81.6)	647 (81.2)	0.919
On-site follow-up care	83 (72.8)	610 (76.5)	0.383
Community agencies	51 (44.7)	258 (32.4)	0.009

Note. STI = sexually transmitted infection; PEP = post exposure prophylaxis;

--Data suppressed due to small cell size.

### Satisfaction with services

Indigenous adult and adolescent women differed significantly from non-Indigenous survivors in their satisfaction with services only in their rating of the overall care provided (Table 6). Indigenous survivors were less likely than non-Indigenous survivors to rate the overall care as excellent or good (95.7% vs. 99.1%, p = 0.045).

**Table 6:** Satisfaction with services of sexually assaulted adolescent and adult women who presented to Sexual Assault/Domestic Violence Treatment Centres, by Indigenous status.

Characteristics	Indigenous n = 72 (%)	Non-Indigenous n = 596 (%)	p-value
	Strongly Agree/Agree	Strongly Agree/Agree	
**Process**			
Timely response to needs	67 (95.7)	542 (92.3)	0.464
Felt safe during visit	69 (97.2)	556 (94.7)	0.565
All questions and concerns responded to	68 (95.8)	558 (94.9)	1.000
Able to choose preferred care	67 (95.7)	555 (94.4)	1.000
Care provided in sensitive manner	69 (97.2)	557 (94.7)	0.565
Satisfied with time staff spent me/us	66 (94.3)	554 (94.2)	1.000
**Provider**			
Treated me/us with respect	69 (97.2)	558 (94.7)	0.565
Supported and cared for me/us	69 (97.2)	556 (94.7)	0.565
Did not judge me/us	69 (97.2)	556 (94.6)	0.567
Believed me/us	69 (97.2)	554 (94.7)	0.565
**Overall**			
Would recommend this service	68 (95.8)	555 (94.5)	1.000
Needed care was received (Yes)*	67 (98.5)	573 (98.8)	0.590
Rating of overall care (Excellent/good)**	67 (95.7)	575 (99.1)	0.045

Note.

*Response option (Yes/No).

**Response options (Excellent/good, Fair/poor).

## Discussion

The majority of the literature available examining sexual assault among Indigenous women focuses on prevalence rates. Our study is the first of which we are aware to explore the presentation, sociodemographic, assailant, assault, and service use characteristics, as well as the service satisfaction of Indigenous adult and adolescent women using specialized violence treatment centres. One hundred and sixteen of 948 women in our sample identified as Indigenous—over 12% of the sample—which is disproportionate to the proportion of Indigenous women and girls in the general female population in Canada (4%) [32]. However, this finding may be explained in part by the heightened rates of violence perpetrated against Indigenous women as compared non-Indigenous women nation-wide [3].

Our study revealed important differences in the presentation characteristics of Indigenous and non-Indigenous sexually assaulted women who were seen at a SA/DVTC. That Indigenous survivors were more likely to come to a hospital within 24 hours of being sexually assaulted is a positive finding as generally this allows for the timely provision of acute health care services, and the collection and documentation of forensic evidence, which can degrade over time [33]. Earlier presentation to a centre among Indigenous survivors is also encouraging given that in Ontario about 40% of Indigenous populations versus 19% of the total Canadian population live in rural or remote areas [34], which is consistent with our finding that Indigenous survivors had an increased likelihood of presenting to centre serving primarily a rural population. The higher rates of police accompaniment to hospital for Indigenous survivors in our study could have contributed to quicker presentation to a centre, as well as alleviated some of the transportation challenges associated with accessing services from rural and remote areas [35]. Other studies have also found that police are more likely to be notified in cases of violent victimizations involving Indigenous women [6, 36–38].

Our study uncovered several important differences in the sociodemographic characteristics of Indigenous and non-Indigenous survivors of sexual assault. Indigenous survivors tended to be younger, a finding congruent with a review of 20 Canadian studies conducted from 1989 to 2007 which found that sexual abuse before the age of 18 is prevalent in Indigenous communities (25%–50%), and more prevalent in Indigenous than non-Indigenous communities [39]. They were also more likely to be living in an institution, which together with younger age, may be related to the increased reporting to police among cases involving Indigenous women. Additionally, Indigenous survivors were more likely than non-Indigenous survivors to be not employed, which may not be surprising given their relatively younger age. At the same time, however, this finding is consistent with the higher rates of unemployment documented for Indigenous versus non-Indigenous women in Canada (13.3% vs. 7.2%) [40]. This profile of Indigenous women in our sample aligns with previous research on the general population of Canadian women that has shown being younger, living in an institutional setting, and being unemployed or having a low income are risks for (re)experiencing sexual assault [41]. Finally, Indigenous survivors were more likely than non-Indigenous survivors to disclose feeling supported by community or group affiliations and government or community services. Although these supports are positive and might mitigate some of the systemic inequalities experienced by Indigenous survivors, there are still many socioeconomic disparities between Indigenous and non-Indigenous women in Canada that urgently need to be addressed by policy makers [42–43].

In our study, we found differences between Indigenous and non-Indigenous survivors on the assailant characteristic, relationship status. The most prominent difference was that Indigenous survivors were more likely to be sexually assaulted by a parent, guardian, or other relative. This finding may be related to Indigenous survivors’ relatively younger age. Furthermore, it aligns with previous reports that have documented high rates of sexual abuse among Indigenous children and youth by members of the immediate or extended family [44–45]. It is important to note, however, that this finding does not suggest that assailants were Indigenous as they could have been a foster parent, parent, family member, or guardian of non-Indigenous heritage. Nonetheless, it is critical in the development of further training for service providers to address the survivor-assailant relationship as familial sexual abuse has been linked to a wide range of adverse psychosocial and behavioral outcomes, including increased likelihood of mental health disorders, particularly depression, substance use, and posttraumatic stress disorder later in life [46–48]. Such training, additionally, could be tailored to better meet Indigenous survivors’ specific needs vis à vis their socioeconomic vulnerabilities.

Some findings from population-based surveys suggest that Indigenous women are more likely to experience violent assaults [3,4]. Our results showed no differences between Indigenous and non-Indigenous survivors in the severity of the assault experienced, including the use of a weapon, use of physical and psychological coercion, and presence of physical injuries. This may be due to the fact that the data examined in our study were hospital-based and therefore already representative of sexual assault cases that were more severe in nature and requiring immediate health care, thus diminishing any potential differences in severity between the two groups. However, compared to non-Indigenous survivors, Indigenous survivors were less likely to report having been forced to drink or having been drugged prior to being sexually assaulted. This finding may be related to the fact that Indigenous survivors were reportedly less likely to be assaulted by non-partner known assailants (e.g., friends, acquaintances), which is the assailant type that has, in one study, been attributed to a substantial proportion of drug-facilitated sexual assault cases [49].

Our study revealed important differences in receipt of services between Indigenous and non-Indigenous sexual assault survivors. Indigenous survivors were more likely to engage in safety planning than non-Indigenous survivors, a finding that is encouraging given the higher rates of violence perpetrated against Indigenous women over their lifetime [3]. Cases involving Indigenous survivors also were more likely to be reported to child protection agencies. Although it must be acknowledged that Indigenous women are problematically over represented among those reported to children’s aid societies in the general Canadian population [50], this finding may simply reflect the younger age of Indigenous survivors and/or the fact that they were more likely to be sexually assaulted by a parent, guardian, or other relative in our sample, circumstances in which reporting could have been mandated [51, 52]. As well, Indigenous survivors were more likely to be referred to agencies in the community for ongoing support, which may suggest fitting receipt of care given some identified community services as a key source of social support.

Notably, our results showed no differences between Indigenous and non-Indigenous women’s receipt of forensic evaluation and acute health care services at SA/DVTCs. This appears appropriate as both Indigenous and non-Indigenous survivors also experienced similar rates of physical coercion, physical injuries, and vaginal and anal penetration. Almost all Indigenous women in our study underwent some component of forensic evaluation (e.g., over four-fifths were assessed for injuries) and used at least one acute health care service (e.g., received crisis counselling, medical care or treatment, HIV post exposure prophylaxis counselling, emergency contraception). These findings coupled with Indigenous survivors’ greater use of safety planning and increased referral to community agencies for ongoing support are somewhat reassuring as the receipt of such services may diminish the short and longer-term physical and psychological impacts of sexual assault, as well as reduce rates of re-victimization [17, 53].

Our results showed that a smaller proportion of Indigenous women rated the overall care provided at SA/DVTCs as excellent or good as compared to non-Indigenous survivors, although this rating of care was high in both groups. Indigenous survivors, like other survivors using the SA/DVTC services, and as seen in other evaluations of specialized sexual assault centres [23, 25], were satisfied with the process and providers of care and almost all agreed that they would recommend the services to others, supporting existing recommendations to further implement SANE-led models of care [16, 54–55]. Given these positive findings and the barriers faced by Indigenous survivors seeking care post victimization, SA/DVTC services should further prioritize outreach initiatives to Indigenous communities [56–57]. As a first step, the Ontario Network of SA/DVTCs is collaborating with the Ontario Federation of Indigenous Friendship Centres, which will lead the creation of relevant tools [58].

### Limitations

The following limitations should be considered in interpreting the results of this study. First, all results from this study are based on women and adolescents attending hospital-based SA/DVTCs. Therefore, some of the findings of this study may not be generalizable to Indigenous and non-Indigenous survivors of sexual assault who choose not to use these services following sexual assault. Second, a substantial proportion of women reported that they did not know or could not remember the details of their assault and thus it is possible that the estimates presented for vaginal and anal penetration could be conservative. Third, there are some data that it would have been valuable to examine in this study that were not deemed feasible or sensitive to collect as part of the Client Evaluation Project acute care environment (e.g., history of childhood trauma). Fourth, survivors who chose not to complete a satisfaction survey may have been those most dissatisfied with services [59]. Fifth, the dichotomization of Likert scales to measure satisfaction resulted in a loss of granulation in the data. However, the a priori decision to analyze the data this way was based on earlier analyses of the larger Client Evaluation Project full sample [19], which revealed very small cell counts in the strongly disagree, disagree, fair, and poor categories. Finally, the Client Evaluation Project was not designed to examine the experiences of Indigenous women specifically and, therefore, relevant items may have not been included on the satisfaction survey (e.g., regarding cultural sensitivity).

## Conclusion

Our key insights into the characteristics and experiences of Indigenous adult and adolescent women seen at Ontario’s SA/DVTCs, as well as their receipt of and satisfaction with services, may have implications for policies and practices at other forensic nurse examiner violence programs, of which there are over 860 globally [60]. Nonetheless, additional studies are needed to expand on our findings. Future research should include larger and more robust examinations of sexual assault using representative samples of Indigenous women in Canada to provide an increasingly accurate picture of the current trends in health and other service use. In order to optimize the post sexual assault care provided to First Nations, Métis, and Inuit women, such studies should extensively and meaningfully engage Indigenous and non-Indigenous service providers, as well as Indigenous survivors themselves, at all stages of the research [61].

## Abbreviations

SANE: Sexual Assault Nurse Examiner
